# Gypenosides, a promising phytochemical triterpenoid: research progress on its pharmacological activity and mechanism

**DOI:** 10.3389/fphar.2025.1705946

**Published:** 2025-10-16

**Authors:** Xue Li, Yiwei Chen, Ruyu Wang, Baorui Cao, Tingting Deng, Jinxiang Han, Meina Yang

**Affiliations:** ^1^ Department of Endocrinology and Metabology, The First Affiliated Hospital of Shandong First Medical University & Shandong Provincial Qianfoshan Hospital, Jinan, China; ^2^ Biomedical Sciences College (Shandong Medical Biotechnology Research Center), NHC Key Laboratory of Biotechnology Drugs, Shandong First Medical University (Shandong Academy of Medical Sciences), Jinan, China; ^3^ College of Traditional Chinese Medicine, Shandong University of Traditional Chinese Medicine, Jinan, China

**Keywords:** Gypenosides, pharmacological activity, antioxidant activity, pharmacokinetics, toxicology

## Abstract

Gypenosides (Gyps), a group of dammarane triterpene saponins that are primarily from *Gynostemma pentaphyllum*, have been identified as promising natural compounds with a diverse array of potent pharmacological activities. In the past 2 decades, a growing body of evidence has demonstrated that Gyps are crucial for the regulation of metabolic homeostasis, the reduction of oxidative stress and inflammation, the protection of the cardiovascular and hepatic systems, and the exhibition of anti-cancer potential. However, obstacles such as limited oral bioavailability, a lack of standardized extracts, and insufficient clinical data restrict the translational potential of Gyps. Recent developments in the pharmacological effects of Gyps, such as the biological characteristics of *Gynostemma pentaphyllum* and the pharmacokinetic and toxicological properties of Gyps, are summarized in this review. We examine the current research limitations and prospective directions for Gyps as potential therapeutic drugs or functional supplements.

## 1 Introduction

**FIGURE 1:**
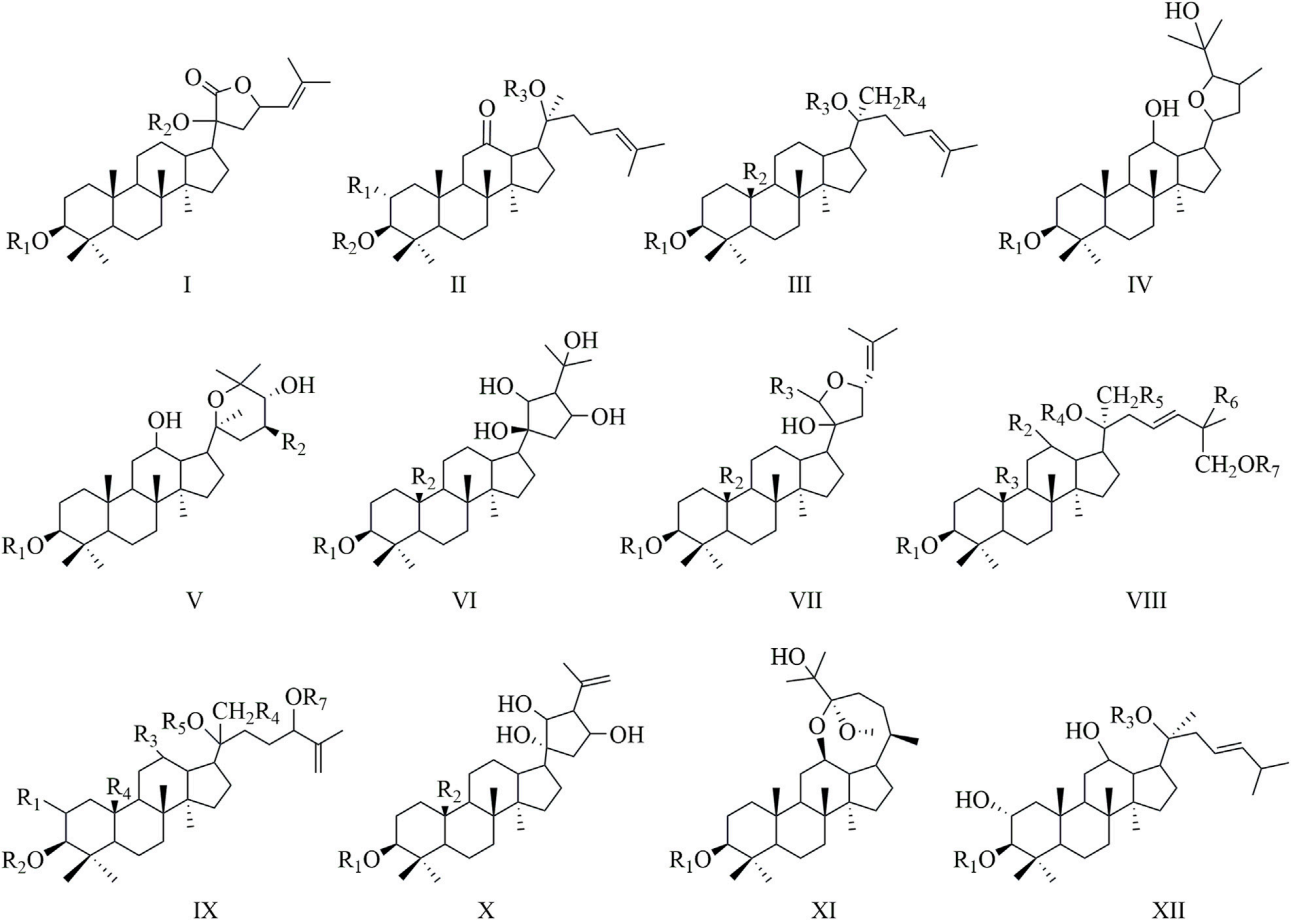
A structural classification of Gyps.

*Gynostemma pentaphyllum* (Thunb.) Makino, or Jiao-Gu-Lan, belongs to the Cucurbitaceae family and has been traditionally employed in East Asian medical practices. The plant’s remarkable adaptability and health advantages have led to its widespread cultivation throughout various regions of China (Liang et al., 2025). The plant contains a diverse array of bioactive metabolites, such as saponins, flavonoids, terpenes, and polysaccharides (Wang et al., 2020b; Chen et al., 2023). The primary active metabolites, referred to as Gyps, are noteworthy for their substantial pharmacological properties, encompassing antioxidant, anti-inflammatory, anticancer, cardiovascular-protecting, and immunomodulatory effects. This highlights their significant therapeutic potential (Pang et al., 2017; Qi et al., 2021; Weng et al., 2021). A taxonomic categorization of Gyps has been conducted, resulting in the identification of 12 separate categories based on their unique structural features (Zheng et al., 2018) (Figure 1). To date, more than 300 distinct Gyps have been found and characterized by *Gynostemma pentaphyllum* (Nguyen et al., 2021).

This review aims to summarize and evaluate recent advancements in research on the therapeutic potential of Gyps, emphasizing their roles in antioxidant, anti-inflammatory, anticancer, and cardiovascular protective mechanisms, among others. Numerous studies have demonstrated that Gyps exhibit significant antioxidant capabilities (Liu et al., 2025). Gyps effectively alleviate oxidative damage by regulating oxidative stress responses and inhibiting the nuclear factor kappa-B (NF-κB) signaling pathway. *In vitro* studies indicate that Gyps enhance the expression of intracellular antioxidant enzymes by upregulating the nuclear factor erythroid 2-related factor 2 (Nrf2) signaling pathway, leading to efficient free radical scavenging and diminished cellular damage (Chen et al., 2022; Ping et al., 2024). Considerable emphasis has been directed towards the therapeutic potential of Gyps in oncological treatment; evidence indicates that Gyps induce apoptosis in cancer cells while inflicting minimal harm to normal cells (Xiao et al., 2024). Furthermore, investigations utilizing animal models have shown that Gyps significantly mitigate symptoms associated with inflammation (Wang et al., 2017b).

**FIGURE 2:**
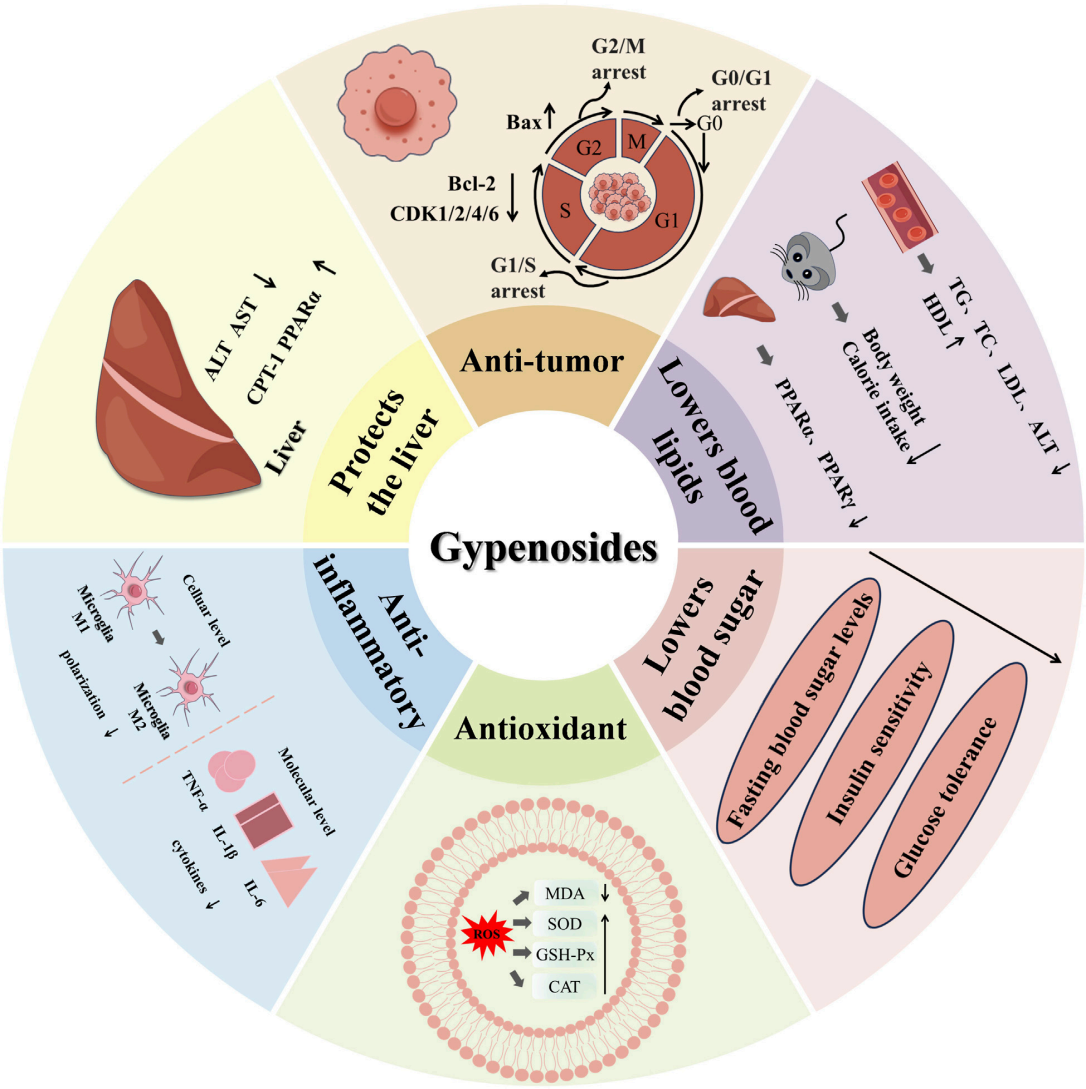
The pharmacological activity and mechanism of Gyps.

Gyps have demonstrated significant cardioprotective qualities in the context of cardiovascular diseases. Gyps are crucial in the prevention and treatment of several cardiovascular diseases by modulating lipid metabolism, reducing blood pressure, enhancing endothelial function, and preventing atherosclerosis. Studies utilizing animal models have demonstrated that prolonged administration of Gyps extract significantly reduces serum cholesterol levels, improves cardiac function, and lowers the incidence of atherosclerosis (Gao et al., 2022). As well, Gyps have been documented to possess hepatoprotective (Song et al., 2017), neuroprotective (Xing et al., 2020), antiviral (Zhao et al., 2022), and other functions (Figure 2).

However, human clinical trials on pure Gyps are extremely limited. A 12-week study in 80 Korean subjects showed that daily supplementation with 450 mg of heat-processed Gynostemma pentaphyllum extract significantly reduced abdominal fat area, body weight, and percentage body fat without adverse effects (Park et al., 2014). Similarly, a 4-week trial (450 mg/day) in 16 healthy men found that Gynostemma pentaphyllum significantly enhanced exercise performance by activating the AMPK pathway (Nayyar et al., 2023). In terms of mental health, anxiety symptoms and stress hormone levels were improved in 72 chronically stressed adults after an 8-week intervention with Gynostemma pentaphyllum 400 mg/d (Choi et al., 2019). In addition, two separate studies on 100 adults confirmed the anti-fatigue (Ahn et al., 2023) and hair health-promoting effects (Lee et al., 2025) of Gynostemma pentaphyllum extract. The small sample size, short research period, and single population continue to limit the application potential of the existing evidence. More rigorous large-scale clinical trials are needed to verify it in the future.

Gyps are natural herbal metabolites of considerable scientific significance and exceptional developmental potential. This review summarizes recent advancements in the pharmacological characteristics, pharmacokinetics, and toxicological profiles of Gyps, as well as the biological qualities of *Gynostemma pentaphyllum*. It provides a comprehensive examination of the antioxidant mechanisms of Gyps, explores their potential therapeutic uses, and aims to establish a scientific foundation for the development of novel therapeutic strategies.

## 2 Biological characteristics of *Gynostemma pentaphyllum*

### 2.1 Botanical characteristics of *Gynostemma pentaphyllum*

*Gynostemma pentaphyllum* is a herbaceous vine belonging to the Cucurbitaceae family. The slender, branching stems are sulcate and bear leaves usually consisting of five to seven leaflets (range: 3–9). The core leaflet measures 3–12 cm in length and 1.5–4 cm in width, whilst the lateral leaflets are relatively smaller. The species is dioecious; male flowers are organized in a slender, extensively branching panicle, sometimes pubescent at the base and occasionally featuring tiny leaflets. Flowers possess a slender pedicel of 1–4 mm in length. The fruit is a sticky, globose berry that is 5–6 mm across and turns black when it's ready. Its surface is smooth and hairless. Each fruit comprises two inverted seeds that are ovate-cordate, roughly 4 mm in width, featuring a blunt apex, cordate base, flattened profile, and two papillate projections. Flowering transpires from March to November, whereas fruiting occurs from April to December (Yang et al., 2019b).

### 2.2 Bioactive metabolites

*Gynostemma pentaphyllum* comprises various bioactive metabolites, including saponins, polysaccharides, and flavonoids. Saponins are the most extensively studied metabolites, demonstrating a wide range of pharmacological activities (Su et al., 2021). Gyps were categorized chemically using cucurbitane-type triterpene skeletons. Its fundamental structure is the cucurbitane core of C-20β-H and C-21-CH_3_, which undergoes C-20 hydroxylation and C-3 glycosylation to produce various saponins (Gypenoside VII, for instance, is 3-O-β-D-glucose). Gypenoside XVII (C-20β-OH), for instance, is the most prevalent active metabolite in *Gynostemma pentaphyllum*, and the antioxidant activity is strongly correlated with its backbone structure. Gypenoside III, IV, VIII, and XII exhibit structural analogies to ginsenosides Rb1, Rb3, Rd, and F2, respectively. This significant chemical similarity indicates the possibility of comparable pharmacological effects (Zhang et al., 2021c; Chen et al., 2023). Ginsenoside, for instance, has been extensively investigated in the context of cognitive function enhancement and anti-aging, with a particular emphasis on the regulation of the NMDA receptor and SIRT1 pathway (Lou et al., 2021; Chu et al., 2025). In clinical practice, Rg3, a representative metabolite of ginsenoside, has been employed as a tumor adjuvant drug (Li et al., 2016b).

Polysaccharides constitute a significant category of bioactive metabolites. Research shows that *Gynostemma* polysaccharides (GPP) are primarily heteropolysaccharides made up of different monosaccharides, with galactose identified as the most prevalent unit (Ji et al., 2018; Wang et al., 2019). Flavonoids, primarily in the form of flavonols and their glycosidic derivatives, represent a notable category of phytochemicals in this plant (Zhao et al., 2024). Additionally, trace amounts of sterols and organic acids have been identified (Li et al., 2016a).

### 2.3 Nomenclature of *Gynostemma pentaphyllum*

*Gynostemma pentaphyllum* is a traditional Chinese medicinal botanical drug obtained mostly from the dried aerial parts of the plant. This plant is widespread in China, South Korea, Japan, and other Southeast Asian areas and is considered a form of “South Asian ginseng” in China (Zhang et al., 2022). The leaves, roots, and stems of *Gynostemma pentaphyllum* are utilized as botanical drugs and in the production of various food items, such as tea, drinks, and biscuits; they are extensively incorporated into daily life (Wang et al., 2020b). Comprehensive pharmacodynamic research demonstrates that *Gynostemma pentaphyllum* possesses various advantageous pharmacological effects, including hypoglycemic, hypolipidemic, anti-cancer, anti-inflammatory, cardioprotective, and neuroprotective capabilities (He et al., 2019; Li et al., 2023d; Jo et al., 2024).

### 2.4 Extraction methods and quality control

Conventional techniques employed to extract the active metabolites of *Gynostemma pentaphyllum* encompass solvent-comparison extraction, ultrasonic-assisted extraction, microwave-assisted enzymatic extraction, and microwave-ultrasonic dual-assisted extraction (Ji et al., 2018; Li et al., 2022a). Methods for the qualitative or quantitative assessment of Gyps encompass thin-layer chromatography, UV spectrophotometry, and high-performance liquid chromatography, among others (Li et al., 2023b).

*Gynostemma pentaphyllum* has not been incorporated into the national pharmacopoeia but is cited in particular regional standards. In 2021, Wen et al. (Xiuping et al., 2021) utilized the testing procedures specified in the general principles of the Chinese Pharmacopoeia (2020 Edition) to assess the quality of *Gynostemma pentaphyllum*. The stipulated parameters mandate that the water content must not surpass 12.0%, the total ash content must not exceed 16.0%, the acid-insoluble ash must remain under 3.0%, and the water-soluble extract content, as ascertained by the hot immersion method, must be at least 25.0%.

## 3 Pharmacological activity of gypenosides

### 3.1 Antioxidant activity

Reactive oxygen species (ROS) are generated by cellular metabolism and external influences. An imbalance between ROS formation and antioxidant defense results in oxidative stress (Hajam et al., 2022). Current research indicates substantial antioxidant capabilities in Gyps. Tables 1, 2 elucidate this activity as recognized at the cellular and organismal levels. Gypenoside XLIX, a principal active metabolite, mitigates oxidative damage caused by several sources. The method entails the suppression of ROS levels while enhancing catalase (CAT), glutathione (GSH), and total antioxidant capacity (T-AOC) (Gao et al., 2022; Xu et al., 2024). Gypenoside XVII (GP-17) similarly provides a protective effect against oxidative damage by decreasing malondialdehyde (MDA) levels in the blood and augmenting the activity of antioxidant enzymes such as superoxide dismutase (SOD), glutathione peroxidase (GSH-Px), and CAT (Yang et al., 2017). Furthermore, in murine asthma models, gypenoside A markedly increases diminished GSH levels while simultaneously decreasing MDA levels, so validating its effectiveness as an antioxidant (Huang et al., 2022a). In rats with atherosclerosis produced by high-fat milk and vitamin D3, the monascus-gypenoside mixture dramatically enhances peroxisome proliferator-activated receptor α and carnitine palmitoyltransferase 1. Consequently, research indicates elevated activities of SOD and CAT in hepatic tissue, accompanied by reduced serum levels of ROS and MDA. A comparative analysis indicates that HG exhibits more antioxidant capacity and improved anti-atherosclerotic efficiency compared to simvastatin (Gou et al., 2018). Additionally, the research indicates that Gyps markedly enhances Nrf2 protein levels, thereby augmenting the downstream expression of heme oxygenase-1 (HO-1) (Ying et al., 2018). These findings indicate that Gyps may confer protection against radiation-induced oxidative damage through the modulation of the Nrf2 antioxidant signaling pathway.

**TABLE 1:** Antioxidant activity of Gyps at animal level.

Active composition and dosage	Models	Findings	Ref.
Gypenoside XLIX (20 mg/kg)	A sepsis-induced ASI model was established in mice	ROS↓, MDA↓, CAT↑ GSH↑, T-AOC↑	Xu et al. (2024)
Gypenoside XLIX (30 mg/kg)	A high-fat choline diet-induced AS model in ApoE−/−mice	MDA↓, SOD↑, GSH-Px↑	Gao et al. (2022)
GP-17 (50 mg/kg)	A high-fat diet-induced AS model in ApoE^−/−^ mice	MDA↓, SOD↑ GSH-Px↑, CAT↑	Yang et al. (2017)
Gypenoside A (10, 30 mg/kg)	An ovalbumin (OVA)-induced asthma mice	MDA↓, GSH↑	Huang et al. (2022a)
The mixture of Hongqu and Gyps (HG) (50, 100, 200 mg/kg)	A high-fat emulsion- and vitamin D3-induced atherosclerotic rats	ROS↓, MDA↓, SOD↑, CAT↑	Gou et al. (2018)
Gyps (50, 100, 200 mg/kg)	The oxidative injury induced by irradiation in mice	MDA↓, SOD↑, CAT↑, GSH↑, T-AOC↑, Nrf2↑, HO-1↑	Ying et al. (2018)

**TABLE 2:** Antioxidant activity of Gyps at cellular level.

Active composition and dosage	Models	Findings	Ref.
Gyps (25, 50, 100, 250, 500 μg/mL)	OFs from Graves’ ophthalmopathy (GO) patients	ROS↓, SOD↑, Nrf2/ERK/HO-1↑	Ma et al. (2022)
Gyps (1, 2.5, 5, 7.5, 10 μg/mL)	Human retinal pigment epithelium ARPE-19 cells	ROS↓, MDA↓, SOD↑ GSH↑, Nrf2↑	Alhasani et al. (2018)
Gyps (50, 100, 200 μg/mL)	H_2_O_2_-Induced Retinal Ganglion Cells	ROS↓, Nrf2↑, ARE↑ OH-1↑	Zhang et al. (2020)
Gyps (80 μg/mL)	H_2_O_2_-Induced Rat Osteoblasts	Oxidative damage to cells↓, NOX4↓	Yanping et al. (2020)
Gyps (5 μg/mL)	Ox-LDL-induced in Human retinal pigment epithelium ARPE-19 cells	oxLDL-induced oxidative stress↓, ROS↓	Biswas et al. (2020)
GP-17 (6.25, 12, 25, 50, 100 μg/mL)	Ox-LDL-induced HUVECs injury model	ROS↓, MDA↓, SOD↑ GSH-Px↑, CAT↑	Yang et al. (2017)
Gyps (50, 100, 150, 200 μg/g and 5 μg/mL)	Cone cells in the rpgrip1 mutant zebrafish	ROS↓, SOD1↑, SOD2↑, GPx1↑, GCLM↑, NQO1↑, Nrf2↑	Alhasani et al. (2020), Li et al. (2021)

ASI, Acute Sepsis-induced Injury; ERK, extracellular signal-regulated kinase; ARE, antioxidant responsive element; NOX4, nicotinamide adenine dinucleotide phosphate-oxidase 4; SOD1, superoxide dismutase 1; SOD2, superoxide dismutase 2; GPx1, glutathione peroxidase 1; GCLM, glutamate-cysteine ligase, modifier subunit; NQO1, NAD (P)H quinone dehydrogenase 1; ARPE-19, adult retinal pigment epithelial cell line-19.

Gyps are vital in combating cellular oxidative stress, especially in ocular cells, where oxidative stress is a major contributor to retinopathy pathogenesis. Gyps has been shown to greatly reduce oxidative stress generated by hydrogen peroxide (H_2_O_2_) in orbital fibroblasts. This reduction is associated with decreased expression of apoptosis-related mRNA and autophagy activation proteins, as well as increased Nrf2/ERK/HO-1 pathway proteins and SOD (Ma et al., 2022). Moreover, Alhasani et al. (2018) discovered that Gyps protects human retinal pigment epithelial cells (RPE) against oxidative damage, which is associated with Nrf2 pathway activation. This method may provide treatment pathways for retinal disorders. Gyps increases antioxidant capacity by increasing Nrf2/ARE and HO-1 expression, inhibits inflammation by downregulating iNOS and COX-2, and reduces apoptosis via the endogenous mitochondrial pathway, protecting retinal ganglion cells (RGCs) from H_2_O_2_-induced damage (Zhang et al., 2020). Recent research shows Gyps protects osteoblasts from oxidative damage caused by H_2_O_2_, in addition to protecting ocular cells. This effect is due to NOX4 downregulation and involves the NOX/BMP/Smad signaling pathway (Yanping et al., 2020). Gyps also decrease oxidative stress generated by oxidized low-density lipoprotein (oxLDL) in RPE cells (Biswas et al., 2020). In particular, GP-17 inhibits ROS and MDA formation while increasing SOD, GSH-Px, and CAT levels in oxLDL-injured human umbilical vein endothelial cells (HUVECs). This chemical activates the ERα-mediated PI3K/Akt pathway, increasing Nrf2 and HO-1 levels and enhancing antioxidant enzymes. OxLDL may reduce HUVEC death by lowering the Bax/Bcl-2 ratio and controlling activated caspase-3—mechanisms previously implicated in retinopathy research (Yang et al., 2017). In addition, Li et al. (Alhasani et al., 2020; Li et al., 2021) discovered that Gyps therapy reduces ROS production, upregulates antioxidant genes (SOD1, SOD2, GPx1, GCLM, NQO1, Nrf2), and increases antioxidant enzyme activity and GSH levels in rpgrip1 mutant zebrafish.

**FIGURE 3:**
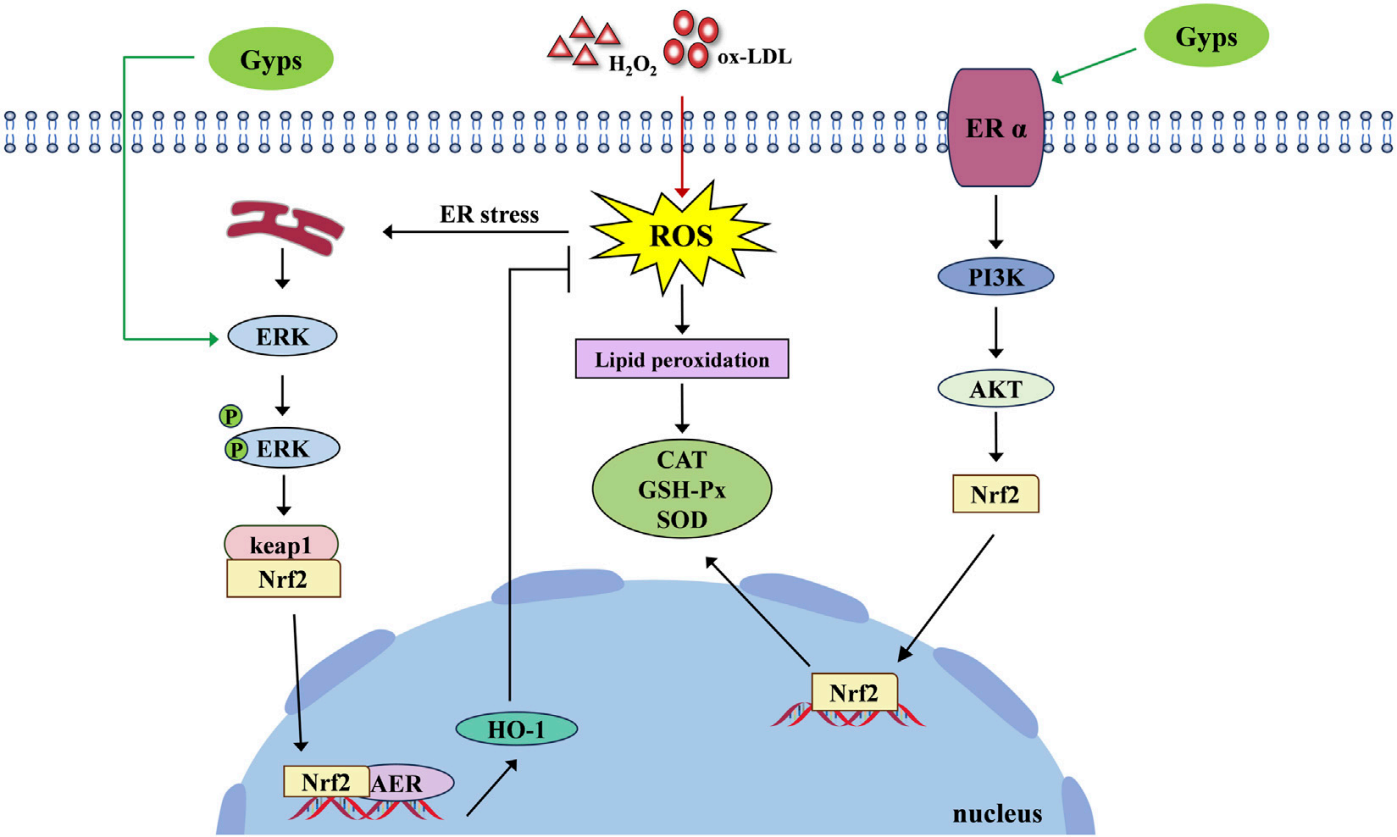
Pharmacological mechanism of the antioxidant activity of Gyps. ER α, estrogen receptor alpha; PI3K, phosphoinositide 3-kinases; AKT, protein kinase B.

While current research has robustly established that Gyps and its monomers exhibit extensive antioxidant properties in cellular and animal models and has preliminarily indicated that Gyps function via critical pathways like Nrf2, significant issues persist in preclinical investigations, including inadequate mechanistic exploration, reliance on singular experimental models, and a deficiency in comprehensive pharmacodynamic and safety assessments. Figure 3 illustrates the pharmacological mechanism of Gyps antioxidant.

### 3.2 Hypoglycemic activity

**FIGURE 4:**
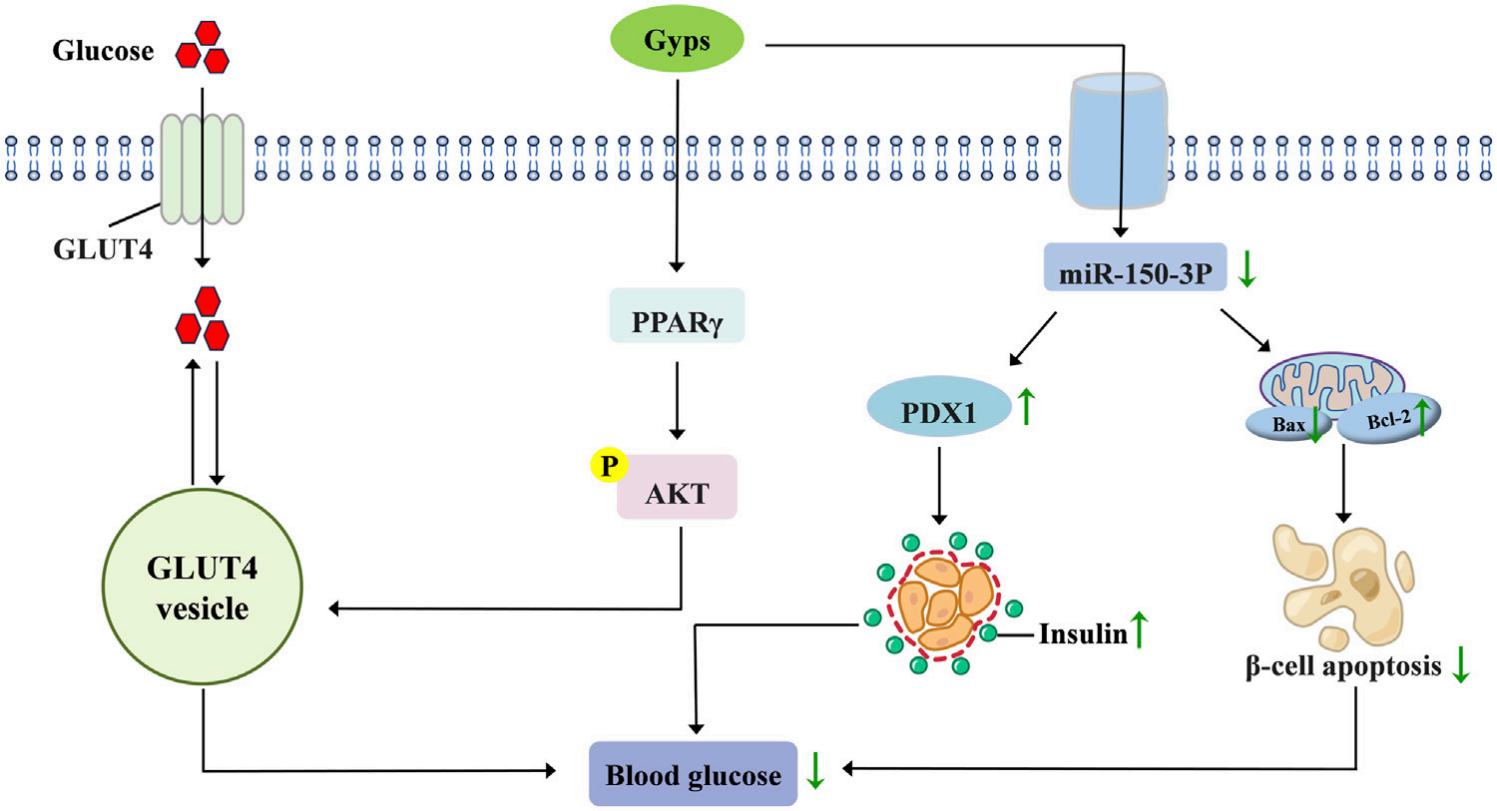
Pharmacological mechanisms of Gyps in lowering blood glucose. GLUT4, glucose transporter type 4; PPARγ, peroxisome proliferator-activated receptor γ; PDX1, pancreatic and duodenal homeobox 1.

Diabetes is among the most prevalent and serious chronic diseases worldwide, characterized by a complex pathogenesis (Chou et al., 2023). GP-75, a natural PPARγ agonist, demonstrates pleiotropic antidiabetic effects. In *db/db* mice models, it significantly reduces fasting blood glucose through time- and dose-dependent mechanisms while enhancing glucose tolerance, insulin sensitivity, and lipid metabolism (Meng et al., 2022). Mechanistic studies reveal that GP-75 activates the PPARγ/Akt/GLUT4 signaling pathway, which enhances cerebral glucose uptake. This action concurrently improves glycemic control (reduced HbA1c, normalized insulin levels) and reverses cognitive impairment (Meng et al., 2023). Regarding pancreatic pathology, gypenoside A ameliorates high-fat diet (HFD)-induced β-cell dysfunction by suppressing miR-150-3p expression, augmenting insulin production, and inhibiting β-cell apoptosis (Li, 2022). Combination therapy employing low-dose berberine, Gyps, and biphenyl diester (BGB) demonstrates synergistic efficacy in mitigating hyperglycemia in T2DM murine models (Zhang et al., 2021a). Gyps attenuate diabetic cardiomyopathy progression by inhibiting ROS-mediated NLRP3 inflammasome activation (Zhang et al., 2018). Collectively, preclinical evidence establishes Gyps as multi-target therapeutic agents that regulate glucose homeostasis, preserve pancreatic function, and prevent diabetic complications. Figure 4 schematically summarizes these hypoglycemic mechanisms.

### 3.3 Hypolipidemic activity

Dyslipidemia is a prevalent group of diseases characterized by elevated levels of atherosclerotic lipids or lipoproteins in plasma or impaired function of anti-atherosclerotic lipids or lipoproteins (Xiang et al., 2022). Gypenoside XIII effectively inhibits hepatocyte lipogenesis and significantly improves hepatic lipid metabolism by blocking fatty acid absorption, accelerating triglyceride breakdown, and reducing hepatic lipid accumulation (Cheng et al., 2024). Concurrently, gypenoside LVI demonstrates regulatory advantages in cholesterol homeostasis, potentially serving as an adjunctive therapy to statins for hypercholesterolemia management (Wang et al., 2021). Thermally processed Gyps activate the SREBP/ACC/PPAR/LXRα signaling axis, reducing serum total cholesterol (TC), triglycerides (TG), and low-density lipoprotein cholesterol (LDL-C) levels while diminishing hepatic lipid accumulation in hyperlipidemic mice (Xie et al., 2023). In obesity models, Gyps reduces body weight and upregulates hepatic PPARα/γ expression (Xie et al., 2022). Mechanistically, Gyps ameliorates high-fat diet (HFD)-induced dyslipidemia by promoting cholesterol-to-bile acid conversion and decreasing the cholic acid/chenodeoxycholic acid (CA/CDCA) ratio (Lu et al., 2018). Lee et al., (2019) confirmed that Gynostemma pentaphyllum extract (GPE) alleviates HFD-induced hyperglycemia and hyperlipidemia in C57BL/6N mice through enhanced AMPK phosphorylation and suppressed lipogenesis without observable toxicity. In terms of intervention for atherosclerosis, Gyps can not only inhibit plaque formation by regulating the PI3K/Akt/bad pathway but also improve vascular disease by lowering the levels of serum adhesion molecules such as ICAM-1, VCAM-1, and MCP-1 (Song et al., 2020; Huang et al., 2022b). While current research has established the potential of Gyps in modulating lipid metabolism and combating atherosclerosis, mechanistic investigations primarily focus on phenotypic associations and lack direct evidence of the regulation of core targets.

### 3.4 Antineoplastic activity

It has been demonstrated that Gyps and their bioactive monomers exhibit significant antitumor activity across a range of cancer types. This provides a theoretical foundation for their development as novel anticancer agents. In lung cancer, both gypenoside L and gypenoside LI have been observed to effectively suppress the proliferation of A549 cells, albeit through distinct mechanisms: gypenoside L primarily induces G0/G1 phase arrest, whereas gypenoside LI triggers G2/M phase arrest (Xing et al., 2018). Recent evidence further suggests that the antitumor effect of Gyps in lung cancer may involve activation of the MAPK14/STAT3 pathway (Qi et al., 2022). In models of breast cancer, gypenoside LI has been observed to promote apoptosis via the upregulation of Bax and the downregulation of PARP-1/Bcl-2 (Zu et al., 2021). In addition, gypenoside I has been shown to inhibit proliferation by suppressing the AKT/GSK3β/β-catenin signaling pathway (Tan et al., 2022).

In renal cancer, gypenoside L and LI have inhibitory effects on cell viability, with Gyps particularly triggering apoptosis via activation of the PI3K/Akt/mTOR pathway (Liu et al., 2022; Liu et al., 2021a). The experiment has shown that gypenoside L inhibits the proliferation of hepatocellular and esophageal carcinoma cells by causing cellular senescence while also augmenting the antineoplastic efficacy of cisplatin and 5-fluorouracil (Ma et al., 2019). Moreover, in melanoma, gypenoside LI has been demonstrated to inhibit tumor development via microRNA-128-3p-mediated cell cycle arrest (Zu et al., 2020). *In vitro* studies further validated the extensive anti-tumor efficacy of Gyps, demonstrating inhibitory effects on several malignant neoplasms, including bladder cancer (Li et al., 2022b), hepatocellular carcinoma (Xiao et al., 2023), and oral squamous cell carcinoma (Lu et al., 2017).

Although existing studies have confirmed the antiproliferative effects of Gyps in various cancers, they have generally overlooked the influence of the tumor immune microenvironment. Furthermore, the drug concentrations used (10–100 μM) significantly exceed clinically achievable blood concentrations. Future research should focus on exploring the regulatory mechanisms of Gyps in the immune microenvironment within pharmacologically relevant concentration ranges to enhance the persuasiveness of its clinical translation.

### 3.5 Anti-inflammatory activity

Inflammation constitutes a complex pathophysiological response orchestrated by diverse pro-inflammatory cytokines and mediators. *In vitro* studies confirm that Gyps extracts enriched with gypenoside XLVI and gypenoside L variably inhibit pro-inflammatory cytokine secretion, demonstrating subtype-specific anti-inflammatory activities among saponin metabolites (Shen et al., 2018). Furthermore, Gyps significantly downregulate mRNA expression of pro-inflammatory mediators (IL-6, IL-1β, COX-2, TNF-α), reduce IL-6 and TNF-α protein levels, and suppress nitric oxide (NO) production, collectively mediating anti-inflammatory effects (Wan and Zhao, 2017; Li et al., 2020; Wang et al., 2020a; Zhang et al., 2020). Notably, GP-17 induces macrophage polarization toward the M2 phenotype and effectively suppresses inflammatory responses in THP-1 macrophage-derived foam cells (Deng et al., 2024). This metabolite exhibits significantly superior anti-inflammatory potency compared to its precursor saponin Rb1 (Zhou et al., 2023a), establishing GP-17 as a promising therapeutic molecule for inflammation modulation.

Under chronic unpredictable mild stress (CUMS) conditions, 7-day Gyps treatment reduced depressive behaviors and hippocampal proinflammatory cytokines (IL-1β, IL-6, TNF-α) in mice (Dong et al., 2018). Gyps significantly suppresses proinflammatory mediators (IL-1β, IL-6, NF-κB) in LPS-induced neuroinflammation and anxiety models, demonstrating therapeutic potential for neuroinflammation-associated anxiety behaviors (Lee et al., 2018). It attenuates optic neuritis in LPS-challenged rats through NF-κB/STAT pathway inhibition (Wang et al., 2018). Oral administration prevents excessive TNF-α/IL-1β production and reduces pathological damage in acute lung injury models (Tu et al., 2021), potentially via STAT-3/HIF-1α and TLR-4/NF-κB/HIF-1α pathway modulation (Xia et al., 2024). Furthermore, GP-17 blocks NLRP3 inflammasome activation and subsequent pyroptosis (Wang et al., 2024). The novel saponin GP-14 specifically inhibits serum IL-6/IL-1β elevation (minimal effect on TNF-α) and exhibits neuroprotective effects against neuroinflammation in high-altitude cerebral edema (HACE) models (Geng et al., 2022). The experimental details of the anti-inflammatory pharmacological activity of Gyps are presented in Table 3.

**TABLE 3:** Experiment of anti-inflammatory activity of Gyps.

Active composition and dosage	Models	Findings	Ref.
Gypenoside XLVI(0–50 μg/mL), Gypenoside L (0–100 μg/mL)	A model of LPS-induced secretion of pro-inflammatory cytokines and mediators in RAW264.7 cells	iNOS↓, IL-6↓, TNF-α↓, COX-2↓	Shen et al. (2018)
Gyps (50, 100, 200 μg/mL)	H_2_O_2_-Induced Retinal Ganglion Cells	COX-2↓, iNOS↓	Zhang et al. (2020)
Gyps (0–250 μg/mL)	LPS-stimulated RAW264.7 macrophage cells	IL-6↓, IL-1β↓, COX 2↓, TNF-α↓	Wang et al. (2020a)
Gyps (10, 25, 50, 100 μM)	IL-1β-stimulated human OA chondrocytes	IL-1β↓, NF-κB↓	Wan and Zhao (2017)
Gyps (25, 50, 100, 250, 500, 1,000 μg/mL)	OFs in Graves ophthalmopathy (GO)	IL-1β↓, IL-6↓ IL-8↓, TNF-α↓	Li et al. (2020)
GP-17 (25, 50, 100, 200 μg/mL)	Ox-LDL-induced THP-1 macrophage	IL-1β↓, IL-6↓, TNF-α↓	Deng et al. (2024)
GP-17 (45–180 μM)	LPS-induced murine RAW 264.7 macrophages	TNF-α↓, IL-6↓	Zhou et al. (2023a)
GP-17 (2.25–9 μmol/kg)	The xylene-induced acute inflammation model of mouse ear edema	TNF-α↓, IL-6↓	
Gyps (25, 50, 100 mg/kg)	CUMS mice model	IL-1β↓, IL-6↓, TNF-α↓, p-NF-κB/NF-κB↓, p-IKKα/IKKα↓ p-IKKβ/IKKβ↓	Dong et al. (2018)
Gyps (25, 50, 100 mg/kg)	A model of chronic inflammation induced by injection of LPS into the rat hippocampus	IL-1β↓, IL-6↓, NF-κB↓	Lee et al. (2018)
Gyps (400 mg/kg)	Lipopolysaccharide-induced optic neuritis model in rats	TNF-α↓, IL-1β↓, COX 2↓, iNOS↓, STAT1↓, NF-κB↓	Wang et al. (2018)
Gyps (100 mg/kg)	LPS-induced ALI mice model	TNF-α↓, IL-6↓, IL-1β↓	Tu et al. (2021)
Gyps (50 mg/kg)	MCAO mice model	IL-6↓,Arg-1↑,CD206↑,IL-10↑, TGF-β↑	Xia et al. (2024)
GP-17 (20, 40, 80 mg/kg)	A mouse bilateral renal ischemia-reperfusion model	IL-1β↓, NLRP3↓, IL-6↓, caspase-1↓, GSDMD↓	Wang et al. (2024)
Gypenoside-14 (100, 200 mg/kg)	A mouse HACE model was established by combinational stimulation with LPS and hypobaric hypoxia exposure	IL-6↓, IL-1β↓	Geng et al. (2022)

iNOS, inducible nitric oxide synthase; IL-6, interleukin-6; TNF-α, tumor necrosis factor-α; COX-2, Cyclooxygenase-2; IL-1β, interleukin-1β; NF-κB, nuclear factor kappa-B; IL-8, interleukin-8; p-NF-κB, phosphorylated NF-κB; IKKα, Inhibitory Kappa B Kinase α; p-IKKα, phosphorylated IKKα; IKKβ, Inhibitory kappa B kinase beta; p-IKKβ, phosphorylated IKKβ; STAT1, Sirtuin 1; Arg-1, Arginase-1; CD206, Mannose Receptor; IL-10, interleukin-10; TGF-β, tansforming growth factor-β; NLRP3, NOD-like receptor thermal protein domain associated protein 3; GSDMD, Gasdermin-D.

### 3.6 Hepatoprotective activity

Non-alcoholic fatty liver disease (NAFLD) represents one of the most prevalent hepatic disorders globally. Without appropriate intervention, it progresses through increasingly severe stages, including non-alcoholic steatohepatitis (NASH), hepatic fibrosis, and ultimately hepatocellular carcinoma (Rong et al., 2023). Gyps modulate the pathological progression of NAFLD through multi-target mechanisms. In NASH, they significantly reduce hepatic triglyceride and free fatty acid accumulation while improving serum activities of alanine aminotransferase (ALT), aspartate aminotransferase (AST), and γ-glutamyltransferase (GGT) (Li et al., 2017; Li et al., 2022a). Gypenoside LXXV specifically attenuates methionine-choline deficient (MCD) diet-induced liver injury, lipid deposition, and macrophage activation (Lee et al., 2020). Against simple steatosis (NAFL), Gyps modulates gut microbiota composition (Huang et al., 2019), inhibits the LPS/Toll-like receptor 4 (TLR4)-mediated inflammatory cascade (Shen et al., 2020), and dually regulates lipid metabolism by suppressing fatty acid/cholesterol synthesis while promoting β-oxidation (Zhou et al., 2023b). Particularly, gypenoside XL alleviates hepatocyte injury through upregulation of peroxisome proliferator-activated receptor α (PPARα) protein expression (Hong et al., 2018). During progression to metabolic dysfunction-associated fatty liver disease (MAFLD), Gyps activates the AMP-activated protein kinase (AMPK) pathway to suppress TLR4/NF-κB signaling, concurrently improving insulin resistance, dyslipidemia (reduced total cholesterol, triglycerides, and LDL-C), and intestinal barrier integrity (Shen et al., 2022). In fibrotic stages, Gyps reduces carbon tetrachloride (CCl_4_)-induced collagen deposition and hydroxyproline content (Hu et al., 2022; Liu et al., 2023b), with gypenoside XLVI demonstrating antifibrotic efficacy in acute/chronic liver injury models (Li et al., 2023c). Notably, gypenoside XLIX ameliorates hepatic steatosis via epigenetic regulation of long non-coding RNA RPARP-AS1 (Liu et al., 2023a) and exhibits cross-disease protection by inhibiting duck hepatitis A virus type 1 (DHAV-1) replication and virus-induced hepatocyte apoptosis (Du et al., 2019). A recent study has indicated that the C3 deglycosylated metabolite of gypenoside XLVI inhibits collagen deposition in liver fibrosis by regulating the AMPK/p300/Smad3 axis in the TGF-β signaling pathway and shows a significant hepatoprotective effect in a mouse model of liver injury (Wang et al., 2025). The experimental details of the hepatoprotective pharmacological activity of Gyps are presented in Table 4.

**TABLE 4:** Experiment of hepatoprotective activity of Gyps.

Active composition and dosage	Models	Findings	Ref.
Gyps (11.49 mg/kg)	A HFD -induced rat NASH models	TG↓, FA↓, ALT↓, AST↓, GGT↓, SREBP-1c↓, ChREBP↓, ACCase↓, SCD-1↓, CPT-1↑	Li et al. (2017)
Gyps (100 mg/kg)	A HFD-induced mouse NASH models	NAS↓, TG↓, ALT↓, AST↓, TG↓, TC↓, LDL-C↓, FBG↓, FINS↓	Li et al. (2022a)
GP-75 (15, 30 mg/kg)	MCD diet-induced mice NASH models	α-SMA↓, TGF-β1↓, TNF-α↓, MCP-1↓, IL-1β↓, NF-κB↓, GRP78↓	Lee et al. (2020)
Gyps (300 mg/kg)	A HFHC-diet induced mice NAFLD models	IR↑, ALT↓, AST↓, TG↓, ACC1↓, PPARγ↓, CD36↓, APOC3↓, MTTP↓	Huang et al. (2019)
Gyps (11.49 mg/kg)	A high-fat diet-induced rat NAFLD models	SOD↑, AST↓, ALT↓, MDA↓, HSI↓, FBG↓, FINS↓, HOMA-IR↓, IL-1Β↓, TNF-α↓, TLR4↑, LPS↑, MyD88↑, p-IκBα↑, p-p65↓, IκBα↓	Shen et al. (2020)
Gyps (250 mg/kg)	A high-fat diet-induced mice NAFLD models	ALT↓, AST↓, TG↓, LDL-C↓	Zhou et al. (2023b)
Gypenoside XL (10, 20 mg/kg)	Choline-deficiency amino acid-defined diet-induced mice NAFLD models	PPARα↑, ACO↑, CPT-1↑	Hong et al. (2018)
Gyps (300 mg/kg)	A HFD/HF- induced rat MAFLD models	HOMA-IR↓, TG↓, TC↓, LDL-C↓, ALT↓, AST↓, TNF-α↓, IL-6↓, IL-1β↓	Shen et al. (2022)
Gyps (100 mg/kg)	Liver fibrosis was induced in rats by CCl4/2-AAF.	Col-I↓, Col-IV↓, α-SMA↓	Hu et al. (2022)
Gyps (3, 10, 30 mg/kg)	The CCl_4_-induced liver damage mouse model	ALT↓, AST↓	Liu et al. (2023b)
Gypenoside XLVI (25, 50 mg/kg and 3, 10, 30 mg/kg)	CCl_4_-induced hepatic fibrosis in mice	AST↓, ALT↓, HYP↓, TNF-α↓, IL-1β↓	Li et al. (2023c)

FA, fatty acid; GGT, gamma glutamyl transpeptidase; SREBP-1c, sterol regulatory element binding protein-1c; ChREBP, carbohydrate response element binding protein; ACCase, acetyl-CoA, carboxylase; SCD-1, stearyl coenzyme a dehydrogenase-1; CPT-1, carnitine palmitoyltransferase1; NAS, non-alcoholic fatty liver disease (NAFLD) activity score; TC, total cholesterol; LDL-C, low-density lipoprotein cholesterol; FBG, fasting blood glucose; FINS, fasting insulin; α-SMA, α-smooth muscle actin; TGF-β1, transforming growth factor-β1; MCP-1, monocyte chemotactic protein-1; GRP78, glucose-regulated protein 78; HOMA-IR, homeostatic model assessment for insulin resistance index; ACC1, acetyl coenzyme a carboxylase 1; PPARγ, Peroxisome Proliferator-activated Receptor-γ; CD36, platelet glycoprotein 4; APOC3, apolipoprotein C3; MTTP, microsomal triglyceride transfer protein; HSI, hepatopancreas somatic indices; TLR4, Toll-like receptor 4; LPS, lipopolysaccharide; MyD88, myeloid differentiation primary response gene 88; p-p65, phosphorylated P65; ACO, acyl coenzyme a oxidase; Col-I, Collagen I; Col-IV, Collagen IV; AHYP, N-acetyl-L-hydroxyproline.

### 3.7 Neuroprotection

Neurodegenerative diseases are chronic progressive disorders characterized by neurodegeneration and neurological dysfunction (Xu et al., 2022). Research has demonstrated that Gyps protects visual function by inhibiting demyelination and axonal degeneration in experimental optic neuritis and neurodegenerative models (Zhang et al., 2017). As demonstrated by Xing et al. (2020), the discovery of novel dammarane saponins, comprising three newly identified structural metabolites along with gypenoside LVII, has shown protective activity against H_2_O_2_-induced oxidative damage in SH-SY5Y neuroblastoma cells. Subsequent research has confirmed that four neodammarane saponins provide concentration-dependent neuroprotection against H_2_O_2_ toxicity without causing cell death in A549 or HepG2 lines (Zhai et al., 2021). Mechanistically, *Gynostemma pentaphyllum* ethanol extract (GP-EX) inhibits α-synuclein-induced dopaminergic neuron death in A53T transgenic mice via ERK1/2-BadSer^112^-JNK1/2 axis regulation (Park et al., 2020). In MPTP-induced Parkinson’s models, Gyps (50 mg/kg) ameliorates memory deficits while restoring tyrosine hydroxylase-positive cells and dopamine levels in substantia nigra/striatum and reactivating hippocampal ERK1/2-CREB phosphorylation (Zhao et al., 2017). Gyps has been shown to provide a substantial neuroprotective impact against hypoxia-induced damage to PC12 cells and to improve the hypoxia tolerance of C57 BL/6 mice when supplied orally (Wang et al., 2022a). In addition, Geng et al., (2022b) discovered that GP-14 pretreatment reduced hypoxia insult to PC12 cell viability and death by activating the AKT and ERK signaling pathways. Studies have indicated that GP-17 can prevent neuronal apoptosis and inflammation, increase functional recovery, and protect against spinal cord damage. This impact is due to upregulation of miR-21, which reactivates the PTEN/AKT/mTOR pathway (Sun et al., 2021). According to a recent study (Lei et al., 2023), gypenoside IX inhibits Aβ synthesis via Akt/GSK-3β signaling, preventing cognitive deterioration.

### 3.8 Other pharmacological activities

Gyps demonstrate renal protective effects and antagonize ischemia-reperfusion (I/R) injury through distinct mechanisms. For renal pathology, they inhibit PI3K/AKT signaling via miR-378a-5p upregulation, thereby reducing TGF-β1-induced fibrosis (Zhang et al., 2024). Gypenoside XLIX directly suppresses TGF-β/Smad3 transduction to attenuate collagen deposition, with its PLGA nanoformulation significantly enhancing antifibrotic efficacy (Liu et al., 2021b). Against I/R injury, this monomer activates cellular survival pathways by disrupting IGFBP7/IGF1R binding, consequently inhibiting renal tubular cell apoptosis and inflammation (Yang et al., 2021). In myocardial protection, gypenoside A mitigates cardiac injury through AMPK pathway activation and miR-143-3p downregulation (Chang et al., 2020), while GP-17 co-activates PI3K/AKT and p38 MAPK pathways to suppress endoplasmic reticulum stress and mitochondrial dysfunction, thereby enhancing functional recovery (Yu et al., 2021).

## 4 Pharmacokinetics of gypenosides

Gyps face significant challenges in oral administration, mainly stemming from poor absorption and low bioavailability, attributed to their high molecular weight, increased polarity, and limited lipid solubility. Studies indicate that the oral bioavailability of total Gyps is approximately 1.2% (Xu et al., 2017; Wang et al., 2022b). A notable absorption pathway involves the metabolism mediated by gut microbiota, which converts Gyps into aglycone forms that possess reduced polarity and enhanced lipid solubility, facilitating absorption across intestinal membranes (Han et al., 2023). The liver is essential for Gyps metabolism and distribution, with evidence showing that bile acid metabolism is regulated by FXR receptor-mediated signaling, which provides hepatoprotective effects (Xie et al., 2020). A validated UPLC-MS/MS method was utilized to quantify gypenoside A and XLIX in rat plasma, revealing notably short half-lives and low bioavailability (0.90% and 0.14%, respectively) (He et al., 2022). Gypenoside A demonstrates rapid absorption, detectable in plasma within 15 min post-administration, achieving peak concentration at 0.75 h, followed by rapid elimination (Hu et al., 2020). Deglycosylation serves as the main metabolic pathway for gypenoside LVI, correlating with reduced intestinal absorption (Chen et al., 2015). Following intravenous injection, GP-17 exhibits rapid elimination, characterized by a half-life of under 2 h. In contrast, oral administration results in swift absorption, achieving peak concentration at 0.19 h. The rapid absorption is likely due to high polarity and low lipid solubility, contributing to its low oral bioavailability of 1.87% (Wang et al., 2017a).

Gyps is rapidly degraded and metabolized *in vivo*, particularly after oral administration, necessitating large or frequent doses. To address this, a tumor-targeted Gyps nanodrug delivery system was developed with 57.64% encapsulation efficiency. However, in artificial intestinal fluid (pH 6.8), its encapsulation efficiency was significantly reduced compared to controls. The SYL3C-Lipo@Gyps-MSN formulation showed minimal drug release (<10%) within 2.5 h, followed by sustained release, enhancing tumor-site drug accumulation and demonstrating suitability for oncological applications (Lai et al., 2024). Incorporating sodium glycocholate (SGC) into nanostructured lipid carriers (NLCs) improved Gyps delivery, achieving 74.22% encapsulation efficiency and 4.89% drug loading (Gyps-SGC-NLCs). *In vitro*, Gyps-SGC-NLCs exhibited sustained release over 48 h, surpassing the Gyps physical mixture (32.2% cumulative release), and increased bioavailability 8.5-fold compared to Gyps powder (Yang et al., 2019a). Encapsulating gypenoside A in mPEG-PLGA nanoparticles yielded 84.4% encapsulation efficiency and 4.02% drug loading, with prolonged release kinetics and enhanced bioavailability versus free gypenoside A (Chen et al., 2024). Similarly, PLGA nanoparticles loaded with gypenoside XLIX achieved 82.4% encapsulation efficiency and 9.04% drug loading, enabling kidney-targeted delivery and sustained release (Liu et al., 2021b).

## 5 Toxicology of gypenosides

Current evidence suggests that human safety data for Gyps are limited to extracted preparations (Shaito et al., 2020). However, there is a lack of hepatotoxicity studies of pure Gyps, and a safe dose range has not been established. Chiranthanut et al., (2013) conducted a study in which rats received 240 mg/kg of *Gynostemma pentaphyllum* aqueous extract via oral gavage for 30 days, resulting in no observed toxicity or mortality. In a comparable study, the daily administration of 5,000 mg/kg of a standardized extract for 90 days demonstrated no observable toxicity. The findings validate the safety of Gyps, even at higher doses. Long-term studies in Wistar rats demonstrated no toxicity or mortality following 6 months of daily administration of 750 mg/kg *Gynostemma pentaphyllum* water extract. No significant changes were observed in body weight, hematological parameters (such as white blood cell count and hemoglobin), hepatic and renal function, or urinalysis outcomes at a dosage of 500 mg/kg/day over the same period. Histopathological examination of key organs (heart, liver, spleen, lungs, and kidneys) revealed no lesions, thereby supporting the safety of extended use (Attawish et al., 2004). Gyps may influence dyslipidemia through the modulation of gut microbiota metabolites, specifically trimethylamine (TMA) and trimethylamine-N-oxide (TMAO); however, the potential chronic risks associated with these metabolites require further investigation (Zhang et al., 2021b). In murine models, a 6-week oral administration of gypenoside L improved exercise capacity and demonstrated anti-fatigue effects (Kim et al., 2022). A 12-week randomized, double-blind, placebo-controlled trial demonstrated that a gypenoside L-containing extract alleviates exercise-induced fatigue without adverse effects, underscoring its therapeutic potential (Ahn et al., 2023). Acute (0.8 g/kg) and subacute (10–50 mg/kg) toxicity studies of GP-75 in mice demonstrated no instances of mortality or significant toxicity (Wu et al., 2024). In randomized controlled trials, the incidence of adverse events of Gyps was substantially lower than that of conventional lipid-lowering drugs, as indicated by the most recent systematic review. Additionally, no serious adverse reactions were observed after continuous use of Gyps for more than 8 weeks (Dai et al., 2022). Gyps demonstrate a favorable safety profile at recommended doses; however, the risks linked to long-term use necessitate further research.

## 6 Conclusions and views

Gyps, the principal bioactive metabolites of *Gynostemma pentaphyllum*, have garnered significant interest owing to their diverse pharmacological actions, including antioxidant, hypoglycemic, anti-tumor, hepatoprotective, and neuroprotective effects. Despite numerous preclinical studies validating their therapeutic promise, three significant hurdles impede clinical translation. Primarily, the majority of saponin monomers have low oral bioavailability and an ambiguous active state *in vivo*, attributable to inadequate membrane permeability and intricate metabolic processes. The second issue is the absence of a defined extraction procedure, leading to irregular metabolite composition; third, variations in plant sources and processing methods result in fluctuations of active compounds. Future study should concentrate on creating novel delivery strategies to enhance bioavailability, instituting standardized quality control measures, and methodically comparing the compositional attributes of saponins from various sources. This will establish the groundwork for achieving the clinical transformation of Gyps and their advancement as lead compounds or adjunct medicinal agents.
